# Supervised oral protein supplementation during dialysis in patients with elevated C-reactive protein levels: a two phase, longitudinal, single center, open labeled study

**DOI:** 10.1186/s12882-015-0070-0

**Published:** 2015-06-23

**Authors:** Srinivasan Beddhu, Rebecca Filipowicz, Xiaorui Chen, Jill L Neilson, Guo Wei, Yufeng Huang, Tom Greene

**Affiliations:** VA Healthcare System, Salt Lake City, UT USA; Department of Medicine, University of Utah School of Medicine, 85 North Medical Drive East, Room 201, 84112 Salt Lake City, UT USA

**Keywords:** Hemodialysis, Inflammation, Protein supplement, Quality of life

## Abstract

**Background:**

Inflammation is considered one of the major causes of protein-energy wasting in maintenance hemodialysis (MHD) patients. It is unclear whether dietary interventions can impact nutritional status and quality of life in MHD patients with elevated C-reactive protein (CRP) levels. Therefore, we examined the hypothesis that supervised intra-dialysis protein supplementation in MHD patients with elevated plasma CRP will improve protein stores and quality of life.

**Methods:**

A 24 week, two phase, longitudinal, single center, open labeled study of 50 MHD patients with plasma CRP > 3 mg/L was conducted. During the 12-week observation phase dietary advice was provided to increase protein intake to 1.2 g/kg/day. In the 12-week treatment phase 45 g of liquid protein supplement was provided at each dialysis treatment. Protein nitrogen appearance (PNA), mid-arm muscle circumference (MAMC), serum albumin, body mass index (BMI) and quality of life (assessed by Short Form-12 questionnaire) were measured at baseline, 12 and 24 weeks.

**Results:**

Median plasma CRP at baseline was 16.0 (IQR 7.7 to 25.1) mg/L. The mean MAMC was 26.5 ± 3.9 cm, BMI 29.2 ± 6.9 kg/m^2^ and plasma albumin 3.8 ± 0.3 g/dl. During the intervention period, mean PNA increased by 0.13 g/kg/d (*p* = 0.01) under a mixed effects model. However, there were no clinically or statistically significant effects on MAMC (*p* = 0.87), plasma albumin (*p* = 0.70), BMI (*p* = 0.09), physical (*p* = 0.32) or mental (*p* = 0.96) composite scores.

**Conclusions:**

In MHD patients with elevated plasma CRP but otherwise mostly normal nutritional parameters, intra-dialytic oral protein supplement was effective in increasing protein intake but did not provide a detectable impact on nutritional status or quality of life.

**Electronic supplementary material:**

The online version of this article (doi:10.1186/s12882-015-0070-0) contains supplementary material, which is available to authorized users.

## Background

The one and three year survival probabilities of incident hemodialysis patients are dismal at 74 % and 50 %, respectively [1]. Protein-energy wasting (PEW) is prevalent in maintenance hemodialysis (MHD) patients, and is by far one of the strongest risk factor for poor outcomes and death in this population [2, 3]. Anorexia and hypercatabolism induced by inflammation is widely considered the underlying cause of PEW in MHD patients [4]. However, it is unclear whether protein supplementation affects body composition, body size and quality of life in MHD patients with elevated C-reactive protein (CRP) levels. Therefore, in an open labeled interventional trial, we examined the hypothesis that supervised protein supplementation during dialysis in MHD patients with elevated serum CRP will improve protein stores (as measured by mid-arm muscle circumference (MAMC) and plasma albumin), body size (as measured by body mass index (BMI)) and quality of life (as measured by Physical and Mental Composite Scores calculated from Short Form-12 (SF-12) questionnaire).

## Methods

This study was conducted between November 13, 2009 and December 18, 2010. The study protocol was approved by the University of Utah IRB. All the participants gave written informed consent.

Adult men and women (age ≥ 18 years) who were on hemodialysis for at least 3 months with serum CRP > 3 mg/L and urea reduction ratio > 65 % who gave informed consent to participate in the study were included. Exclusion criteria were patients who were unable to give informed consent, prisoners or pregnant women, current active malignancy (excluding squamous and basal cell carcinoma), active AIDS, and cirrhosis/active liver disease with poor prognosis.

### Study design and procedures

This study was designed as a 24-week, two phase, longitudinal, single center, open labeled study using convenience sampling. Predialysis blood was obtained for measurement of plasma high-sensitivity CRP (hsCRP) and those with levels > 3 mg/L were included in the study. During the observation phase, all participants received dietary advice to increase protein intake to 1.2 g/kg/day and were monitored for 12 weeks. If they were already on a protein supplement they were advised to continue that supplementation during the observational phase.

During the treatment phase, in addition to the protein supplementation if any that the participant was on, supervised supplementation on each dialysis session was provided. Each participant received 45 g of liquid protein supplement (Provide Sugar Free produced by Provide Nutrition LC) at each dialysis treatment for additional 12 weeks for total study duration of 24 weeks. Nutrition content information of the supplement is provided in the Additional file 1: Appendix Table 4 and Appendix Figure 1 [5].

### Clinical and anthropometric measurements

Anthropometric measurements were obtained at baseline, 12 and 24 weeks following standardized protocols by trained renal dieticians. Triceps skin fold thickness was measured with Lange calipers at the back of the arm at the halfway point between the olecranon process of the ulna and the acromion process of the scapula with the person standing upright and arms hanging down loosely. The skin fold was pulled away from the muscle and measured with the calipers, taking a reading 4 s after the calipers have been released. At the same point, mid-arm circumference was measured with a non-stretchable tape measure. MAMC in cm was calculated as mid-arm circumference (cm) - (0.314 x triceps skinfold in mm) [6]. Patients’ weight, height, and waist circumferences were obtained predialysis during midweek sessions (Wednesday for MWF, Thursday for TuThSat). Post-dialysis weight was also measured and BMI was calculated as post-dialysis weight divided by height squared (in kilograms per square meter).

Quality of life was measured at baseline, 12, and 24 weeks using the SF-12, a validated instrument assessing general health employing a Physical Component Summary (PCS) and the Mental Component Summary (MCS) [7]. Mid-week pre and post dialysis blood samples were drawn in lithium heparin tubes at baseline, 12, and 24 weeks. The blood sample was centrifuged within 15 min and the plasma divided into multiple aliquots of 1 ml each and frozen immediately with dry ice and transferred to a −80 °C freezer. In those who reported having a urine output of at least 1 cup/day, 44 h urine was collected from the end of first dialysis treatment of the week to the beginning of the mid-week dialysis at baseline, 12 and 24 weeks. Participants were instructed to store the urine samples in a cold place and bring it with them to the dialysis unit. 1.8 ml of urine sample was aliquoted in a tube and was transported from the dialysis unit to the −80 °C freezer on dry ice. hsCRP was measured using a latex-particle enhanced immunoturbidimetric assay kit (Roche Diagnostics, Indianapolis, IN 46250) and read on the Roche Modular P Chemistry analyzer (Roche Diagnostics) at the University of Utah Associated Regional University Pathologists (ARUP) Laboratory. Pre and post dialysis blood urea nitrogen (BUN) and urine urea nitrogen were measured using the Roche Modular P Chemistry analyzer (Roche Diagnostics) at the ARUP Laboratory. interleukin 6 (IL6) and tumor necrosis factors α (TNFα) were measured using DuoSet ELISA development system (Quantikine; R & D Systems Inc.; Minneapolis, MN, USA).

### Assessment of adherence

The amount of supplement consumed during each treatment was recorded by the dialysis unit personnel. Achieved protein supplement dose was calculated from the amount of protein supplement consumed. In addition, protein nitrogen appearance (PNA) calculated [8] from mid-week pre and post-dialysis BUN levels using a two-BUN measurement, single-pool, variable volume model as PNA = C_0_/(25.8 + (1.15/Kt/V) + (56.4/Kt/V)) + 0.168 where, C_0_ is predialysis BUN and Kt/V is dialysis clearance. Kt/V was calculated as Kt/V = -Ln (R-0.008xt) +4-(3.5x R)) x UF/W where R is the postdialysis/ predialysis BUN ratio, t is the dialysis session in hours, UF is the ultrafiltration volume in liters, and W is the postdialysis weight in kilograms [8]. In those with residual renal function, C_0_ was upwardly adjusted using the formula C_o_’ = C_o_ [1 + (0.79+ (3.08)/(Kt/V)) Kr/V], where Kr is residual urinary urea clearance in mL/min, C_0_’ and C_0_ are in mg/dL, and V is in L [8].

### Statistical analysis

Baseline clinical characteristics, protein supplementation and plasma inflammatory markers levels at baseline and follow-up were summarized using means and standard deviations or by medians and 25^th^ and 75^th^ percentiles for continuous variables and by proportions for categorical variables. The effects of the treatment on outcomes were evaluated primarily by the mean changes in the outcome variables during the 12-week interventional phase. Secondary analyses evaluated the difference between the mean changes of the outcomes during the 12-week interventional phase and the mean changes during the 12-week observational phase. The latter assessment evaluates the change in the mean slope of the outcomes between the interventional and observational changes, and is intended to correct for long-term trends in the outcomes which are independent of the intervention. The mean changes in both the observational and interventional phases were estimated by applying mixed effects analyses using an unstructured covariance model to account for correlations in measurements across time. These models were used to characterize mean changes in PNA, MAMC, plasma albumin, BMI, PCS and MCS. Sensitivity analyses were performed limiting the mixed effects analyses to only those with low baseline PNA (<1 g/kg/d) or low plasma albumin (<4 g/dl) or high plasma hsCRP (>10 mg/L).

Additional analyses were conducted in the entire study population to estimate the mean changes in the outcome variables during the intervention period after adjusting for contemporaneous changes during the intervention period in the pre-dialysis levels of plasma markers of inflammation (hsCRP, TNFα and IL6), plasma bicarbonate and residual renal function. These analyses were carried out by modifying the mixed effects models to relate the mean changes in the outcomes to the changes in each of the indicated covariates during the interventional phase, and reporting the estimated mean change in the outcome when the mean change in each covariate was set to 0.

Analyses of each outcome were performed on a comparison-wise basis, without adjustment for multiple comparisons. We performed analyses using STATA 12 and SAS version 9.2.

Furthermore, in order to descriptively compare the baseline nutritional parameters of this dialysis cohort to the population norms of adults > 50 years of age, we used data from 1999 to 2002 National Health And Nutrition Examination Survey, the details of which are published elsewhere [9]. We used the svy suite in STATA 12 to conduct the National Health and Nutrition Examination Survey (NHANES) analyses.

## Results

**Fig. 1:**
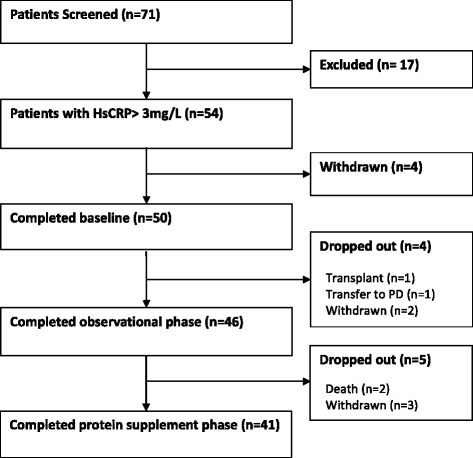
Flow of study participants

The flow of study participants is summarized in Fig. 1. Seventy-one patients underwent screening visit and of these, 54 had serum hsCRP > 3 mg/L and were included. Of these, 50 participants completed baseline visit and started the observational phase. Four participants dropped out during the observational phase. Forty-six participants started the interventional phase and 5 dropped out during that phase. Forty-one participants were able to complete both phases of the study successfully.

Table 1 summarizes the baseline characteristics of the study dialysis population and a national sample of older healthy community dwelling adults. As is expected, the dialysis population had a greater prevalence of comorbidities. In comparison to the healthy older cohort, the dialysis population also had very high hsCRP levels, lower plasma albumin levels and lower muscle mass. Nonetheless, the dialysis population had higher fat energy stores (as evidenced by higher BMI and waist circumference).

**Table 1:** Clinical and nutritional characteristics. Characteristics of the study dialysis population (n = 50) and adults > 50 years of age in the US general population (1999–2002 National Health And Nutrition Examination Survey)

	Current study population (n = 50)	NHANES 99–02 with age ≥ 50 years (n = 4983)^a^
Demographics		
Age (year)	63.8 ± 17.5	64.0 ± 9.2
Women (%)	44.9	54.5
African American (%)	4.1	8.8
Comorbid conditions		
Coronary artery disease (%)	46.9	7.9
Cerebrovascular disease (%)	20.4	5.0
Congestive heart failure (%)	40.8	3.4
Diabetes (%)	66.7	14.6
Malignancy (%)	14.3	15.7
Smoking (%)	40.8	54.2
Nutritional and inflammatory markers		
Body mass index (kg/m^2^)	29.2 ± 6.9	28.5 ± 5.2
Waist circumference (cm)	106.6 ± 16.7	99.5 ± 12.7
MAMC (cm)	26.5 ± 3.9	32.8 ± 4.1
Plasma albumin (g/dL)	3.8 ± 0.3	4.3 ± 0.3
Plasma hsCRP (mg/L)	16.0 (7.7, 25.1)	0.2 (0.1, 0.5)
Dialysis and Renal Characteristics		
Duration of ESRD (years)	3.0 (1.4, 4.8)	NA
AV Fistula (%)	77.6	NA
Kt/V	1.6 ± 0.2	NA
Proportion with urine output > 1 cup/day (%)	34.7	NA

^a^Adjusted for NHANES survey weight

Weekly supervised protein supplementation during the intervention period is summarized in Table 2. At baseline, 12 and 24 weeks, 46 %, 48 % and 46 % of the participants reported taking an unsupervised protein supplementation on their own, respectively. The reported mean unsupervised protein supplement intake levels were 63.8 ± 91.8, 62.2 ± 84.9 and 64.9 ± 96.8 g/week, respectively.

**Table 2:** Weekly average of supervised protein supplementation during dialysis in the interventional period

Week	N	N non-adherent^a^	g/week	g/kg/d
13	46	2 (4.3 %)	118.0 ± 32.2	0.21 ± 0.07
14	45	3 (6.7 %)	117.0 ± 37.6	0.21 ± 0.07
15	45	7 (15.6 %)	106.3 ± 47.4	0.18 ± 0.09
16	45	7 (15.6 %)	101.7 ± 51.6	0.18 ± 0.10
17	45	7 (15.6 %)	95.0 ± 51.7	0.16 ± 0.09
18	44	8 (18.2 %)	94.8 ± 51.9	0.16 ± 0.10
19	44	6 (13.6 %)	108.1 ± 46.4	0.19 ± 0.09
20	44	6 (13.6 %)	107.4 ± 48.7	0.18 ± 0.09
21	42	6 (14.3 %)	103.9 ± 50.2	0.18 ± 0.09
22	42	7 (16.7 %)	105.0 ± 52.3	0.18 ± 0.10
23	42	8 (19.0 %)	97.5 ± 53.4	0.17 ± 0.10
24	41	8 (19.5 %)	86.0 ± 57.1	0.15 ± 0.10

^a^Non-adherence to intervention was defined as consumption of < 50 % of provided protein supplement or continuing in the study after stopping the intervention

**Fig. 2:**
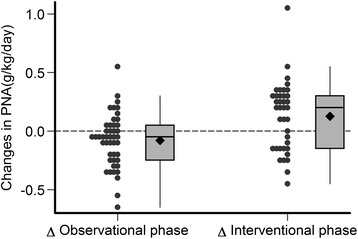
Changes in protein nitrogen appearance during the observational and interventional phases

Table 3 summarizes the measured PNA from urea kinetic modeling, nutritional and quality of life measures, plasma inflammatory markers and bicarbonate levels and residual renal function at baseline, week 12 and week 24. During the observation period mean PNA decreased whereas it increased in the intervention period (Fig. 2). Spearman correlation between the achieved protein supplement between weeks 12 and 24 and the delta of PNA between weeks 12 and 24 was 0.45 (*p* = 0.004).

**Table 3:** Measured protein nitrogen appearance, nutritional and quality of life measures, plasma inflammatory markers and bicarbonate levels and residual renal function at baseline, 12 and 24 weeks^a^

	Baseline N = 50	Week 12 N = 46	Week 24 N = 41
PNA (g/kg/day)	1.12 ± 0.32	1.04 ± 0.30	1.18 ± 0.35
MAMC (cm)	26.5 ± 3.9	26.2 ± 4.0	26.6 ± 4.3
Plasma albumin (g/dL)	3.76 ± 0.31	3.79 ± 0.33	3.75 ± 0.35
Body Mass Index (kg/m^2^)	29.2 ± 6.9	29.0 ± 6.9	28.8 ± 5.6
SF12 - Physical Health Composite Scale Scores	29.3 ± 9.6	30.6 ± 9.9	29.2 ± 9.0
SF12 - Mental Health Composite Scale Scores	51.4 ± 11.3	50.9 ± 11.1	50.6 ± 13.1
Plasma hsCRP (mg/L)	16.0 (7.7, 25.1)	10.9 (6.4, 25.3)	14.0 (7.9, 38.0)
Plasma TNF-α (pg/ml)	15.4 (9.1, 43.0)	17.4 (11.0, 41.8)	19.3 (12.4, 94.4)
Plasma IL6 (pg/ml)	10.7 (5.1, 20.2)	13.2 (8.9, 19.9)	12.8 (5.0, 35.5)
Plasma bicarbonate (mmol/L)	24.2 ± 4.4	26.1 ± 3.1	26.3 ± 3.4
% with residual renal function	34.7	26.1	20.5
24-h urine volume in those with residual renal function (ml/d)	648.0 ± 374.3	562.5 ± 371.6	643.9 ± 331.3

^a^Mean ± SD or median (25^th^, 75^th^ percentiles) presented

In the mixed effects analysis, mean PNA significantly increased by 0.13 (95 % CI, 0.03 to 0.22) g/kg/d during the interventional phase (Table 4, first row). Because PNA declined slightly during the observation period, the change in PNA during the intervention period was 0.21 (95 % CI, 0.08 to 0.33) g/kg/d greater (*p* = 0.001) than during the during the observation period (Table 4). Similar results were obtained after adjusting for changes during the intervention phase in the levels of markers of inflammation, plasma bicarbonate and residual renal function (Table 5).

**Table 4:** Mixed effects models of changes in protein intake and outcome variables

	Δ interventional phase (95 % CI), *p* values	Δ interventional phase – Δ observational phase (95 % CI), *p* values
PNA (g/kg/day)	0.13 (0.03, 0.22), *p* = 0.01	0.21 (0.08, 0.33), *p* = 0.001
MAMC (cm)	0.06 (−0.62, 0.73), *p* = 0.87	0.02 (−1.16, 1.21), *p* = 0.97
Plasma albumin (g/dL)	−0.01 (−0.09, 0.06), *p* = 0.70	−0.05 (−0.16, 0.06), *p* = 0.35
BMI (kg/m^2^)	−0.22 (−0.47, 0.03), *p* = 0.09	−0.24 (−0.66, 0.18), *p* = 0.25
SF12- PCS	−1.39 (−4.15, 1.38), *p* = 0.32	−2.55 (−6.49, 1.38), *p* = 0.20
SF12- MCS	0.07 (−2.70, 2.85), *p* = 0.96	0.74 (−4.46, 5.94), *p* = 0.78

**Table 5:** Additional analysis by mixed effects models^a^ of changes in outcome variables adjusted for plasma levels of markers of inflammation and bicarbonate and residual renal function

	Δ interventional phase^a^ (95 % CI), *p* values
PNA (g/kg/day)	0.17 (0.05, 0.29),*p* = 0.005
MAMC (cm)	0.50 (−0.39, 1.40),*p* = 0.262
Plasma albumin (g/dL)	−0.04 (−0.14, 0.05),*p* = 0.368
BMI (kg/m^2^)	−0.19 (−0.53, 0.16),*p* = 0.282
SF12- PCS	−2.91 (−6.86, 1.04),*p* = 0.145
SF12- MCS	1.18 (−1.77, 4.12),*p* = 0.426

^a^Adjusted for the change in interventional phase in plasma hsCRP, TNFα, IL6 and bicarbonate and residual renal function

**Fig. 3:**
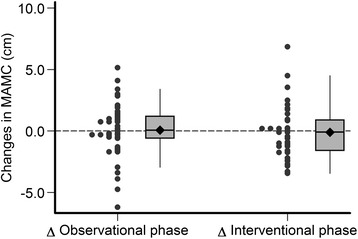
Changes in mid-arm muscle circumference during the observational and interventional phases

**Fig. 4:**
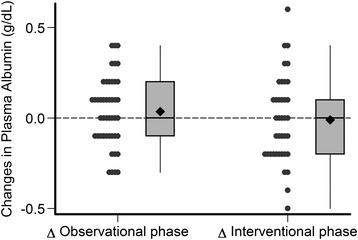
Changes in plasma albumin during the observational and interventional phases

**Fig. 5:**
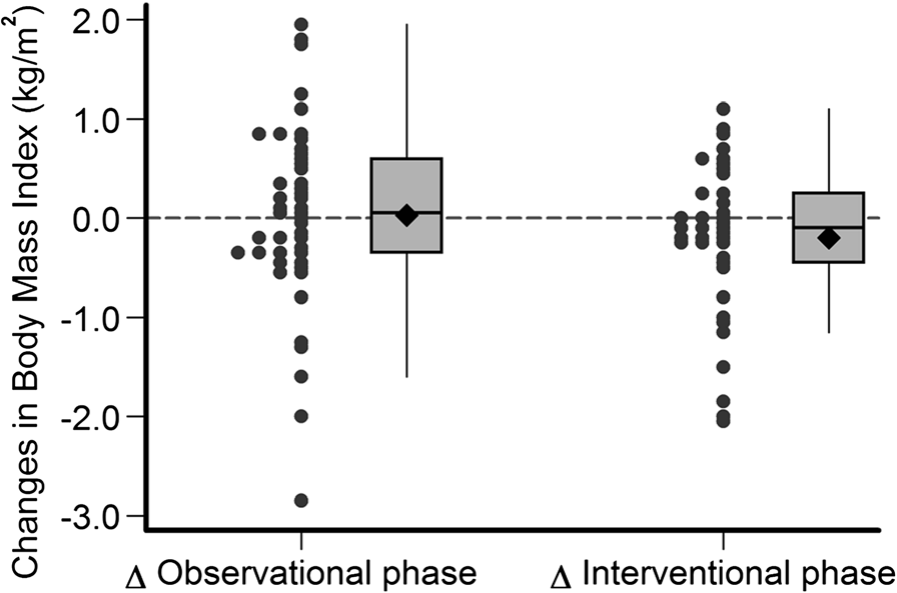
Changes in body mass index during the observational and interventional phases

**Fig. 6:**
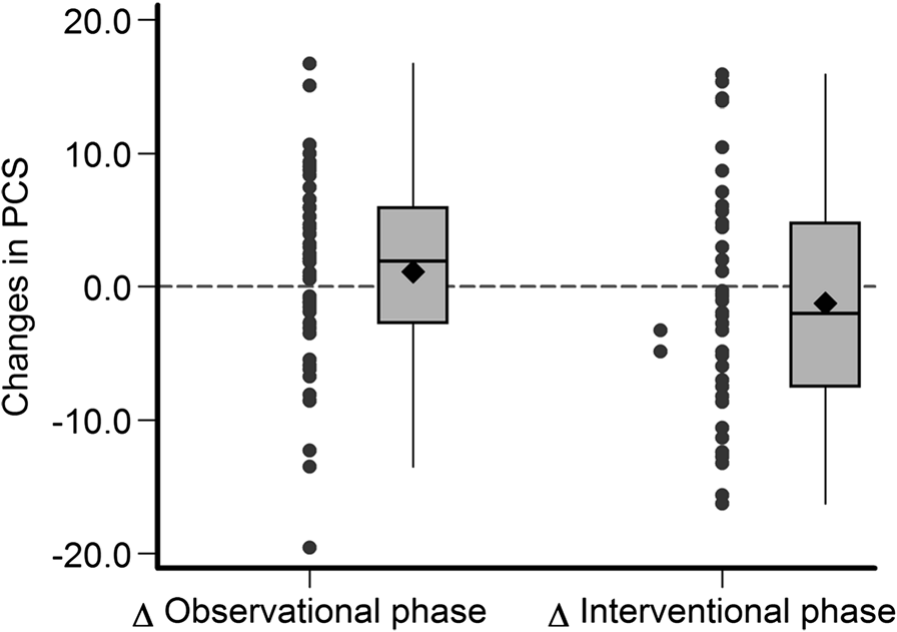
Changes in physical health composite scale scores during the observational and interventional phases

**Fig. 7:**
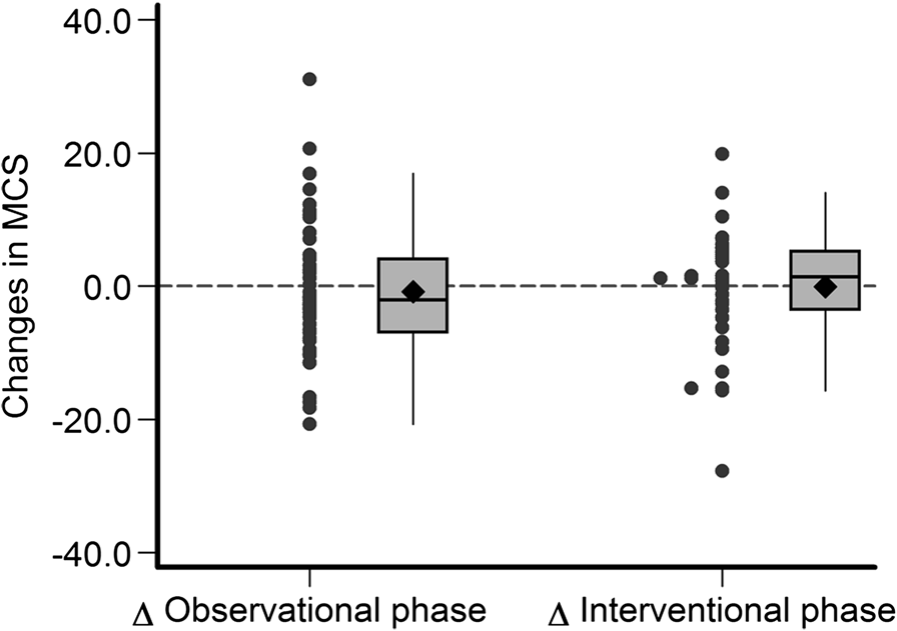
Changes in mental health composite scale scores during the observational and interventional phases

The estimated mean changes of MAMC, plasma albumin, BMI and SF-12 during the observational and interventional phases of the study are provided in Table 4 and in Figs. 3, 4, 5, 6 and 7. Without covariate adjustment, there were no statistically significant mean changes of any of the outcomes during the intervention phase (left columns of Tables 4 and Figs. 3, 4, 5, 6 and 7) and also no statistically significant differences in the changes between the intervention and observational phases (right columns of Table 4). It is noteworthy that the effect sizes for all of the outcome variables were small with relatively narrow confidence intervals suggesting that type 2 error (falsely negative results due to failure to detect clinically important effects of protein supplementation on these parameters) is unlikely.

When further adjusted for plasma markers of inflammation and bicarbonate and residual renal function, the results were similar (Table 5).

The results were also similar in subgroups defined by baseline PNA or plasma albumin or plasma hsCRP (please see Additional file 1: Appendix Tables 1-3).

## Discussion

Seventy-six percent of the screened dialysis patients in this study had plasma hsCRP levels > 3 mg/L indicating that inflammation is highly prevalent in dialysis patients. Compared to the healthy community dwelling older adults, these dialysis patients appear to have lower protein stores (as evidenced by lower serum albumin and lower muscle mass) but higher energy stores (as evidenced by higher BMI and higher waist circumference). Furthermore, in dialysis patients with inflammation, intra-dialytic oral protein supplementation was effective in producing a clinically meaningful increase in protein intake as measured by PNA. However, protein supplementation did not impact on muscle mass, plasma albumin or quality of life in these hemodialysis patients. The following discussion interprets these findings in the context of existing literature.

The current national guidelines recommend a dietary protein intake of 0.8 g/kg/d in the general population and 1.2 g/kg/d in hemodialysis patients [10]. These recommendations are largely based upon observational data that suggest that low protein intake as well as markers of PEW is associated with increased mortality. There has been only one randomized controlled trial that examined the effects of protein supplementation on mortality in dialysis patients. However, that study compared intra-dialytic parenteral nutrition (IDPN) vs. oral protein supplement and did not include an usual care arm [11]. In that study, in both groups PNA increased along with an increase in body weight and serum albumin but there were no differences between the two groups regarding the primary endpoint of mortality [11].

Muscle is the largest protein store in the body. Short term amino-acid labeling studies suggest that protein supplementation could improve muscle anabolism [12]. However, there is surprisingly very little data on the effects of protein supplementation on muscle mass in hemodialysis patients. In an earlier smaller study of 20 dialysis patients, protein supplementation did not impact on muscle mass [13]. In the current study also we did not observe an effect of protein supplementation on MAMC.

Non-randomized and randomized trials that examined the impact of protein supplementation on serum albumin in dialysis patients have yielded mixed results. Some of the trials showed clinically meaningful increases in serum albumin levels [14–16], whereas others showed very modest increases [11, 13, 17–20] or no detectable effects [21]. Two recent large observational studies of oral nutritional supplement suggested a better survival with nutritional supplement [22, 23] however, in one of those studies [22] follow-up serum albumin levels were not available and in the other study [23], no effects of nutritional supplementation on serum albumin was observed. In the current study, we did not note a significant change in plasma albumin with protein supplementation.

The dose of supervised dialysis protein supplementation in this study (45 g/dialysis session) was much higher than the doses of 16.6 g per dialysis session [16] and 31.5 g per dialysis session [20] in earlier studies. The lack of detectable effects on muscle mass and serum albumin despite a clinically meaningful increase in PNA might be ascribed to the high CRP (median 16.0, IQR 7.7 to 25.1 mg/L) levels in this cohort. However, in the study by Cano et al., there was modest increase in serum albumin in those treated with oral/ parenteral protein supplementation in the presence or absence of elevated CRP [11]. Hence, it is unlikely that inflammation is the reason for the negative results in the current study.

It should be noted that the upper bounds of the 95 % confidence interval for treatment effects of MAMC were 0.73 cm, plasma albumin 0.06 g/dl and BMI 0.03 kg/m^2^ (Table 4). The small magnitudes of these upper endpoints suggest that while the possibility of positive treatment effects cannot be ruled out by this study (i.e., the null hypothesis cannot be shown to be true), any such undetected effects are likely to have been relatively small.

Compared to the national norms (Table 1), despite the higher prevalence of comorbidity, higher concentrations of hsCRP, lower concentrations of plasma albumin and lower muscle mass, dialysis patients do not appear to be “wasted” in the sense that they have higher BMI and higher waist circumference. Hence, they do not appear to have lower energy stores. Therefore, the reason for lack of a beneficial effect of protein supplementation in this study on nutritional markers might be because these patients might not need protein supplementation in the first place. Indeed in studies where the BMI was low [11, 15] or serum albumin was low [11, 14, 15], protein supplementation was effective in increasing serum albumin and body weight. Furthermore, in a non-randomized observational study of dialysis patients with serum albumin ≤ 3.5 g/dL, those who received nutritional supplement had better survival compared to those who did not [22]. In other words, despite the current recommendations to increase PNA in all dialysis patients to 1.2 g/kg/d, nutritional interventions might need to be more carefully targeted to those with poor nutrition as evidenced by low BMI or low serum albumin.

Nonetheless, the current guidelines and clinical practice are to target with protein supplementation a PNA of 1.2 g/kg/d and serum albumin of 4 g/dl. Hence, the current study was designed to examine the effects of oral protein supplementation on nutritional markers in inflamed dialysis patients. To our knowledge, this is the first study to examine the impact of protein supplementation on nutritional markers in hemodialysis patients with elevated plasma CRP levels.

The limitations of the study include a lack of parallel arm randomized controlled design, shorter duration and smaller number of participants. Nonetheless, these limitations also apply to most of the above mentioned interventional studies on protein supplementation in dialysis patients. Protein sources rich in branched chain amino acids might be beneficial [24] but this study was not designed to examine that hypothesis. While protein intake was objectively measured with urea kinetic modeling, energy intake was not measured in this study. Finally, BMI, which is based on the height and weight of an individual, is an inaccurate indicator of body composition because it does not take into account muscle mass. We used MAMC as the main measure of muscle mass in this study and we did not obtain other measures such as computed tomography (CT) or magnetic resonance imaging (MRI) scans or dual energy x-ray absorptiometery (DEXA).

## Conclusion

In summary, most dialysis patients have inflammation as defined by elevated CRP levels. Despite, the high CRP levels and lower plasma albumin and MAMC, dialysis patients appear to have higher energy stores. High dose intra-dialytic protein supplementation did not affect MAMC, body weight or plasma albumin in this relatively well-nourished dialysis patients. Interventional studies targeting those with evident malnutrition as defined by lower body size or low muscle mass are needed to define the role of dietary interventions in dialysis patients.

## Additional file

Additional file 1: **Supplemental materials.** **Appendix Table 1** - Effects of protein supplementation on nutritional parameters in sub-groups defined by baseline PNA. **Appendix Table 2** - Effects of protein supplementation on nutritional parameters in sub-groups defined by baseline plasma albumin. **Appendix Table 3** - Effects of protein supplementation on nutritional parameters in sub-groups defined by baseline plasma hsCRP. **Appendix Table 4** - Provide Gold® Sugar Free 30oz bottles amino acids components. **Appendix Figure 1** –Provide Gold® Sugar Free 30oz bottles nutrition facts.

## Abbreviations

MHD: Maintenance hemodialysis
CRP: C-reactive protein
PNA: Protein nitrogen appearance
MAMC: Mid-arm muscle circumference
BMI: Body mass index
PEW: Protein-energy wasting
hsCRP: High-sensitivity CRP
SF-12: Short Form-12
PCS: Physical component summary
MCS: Mental component summary
BUN: Blood urea nitrogen
TNFα: Tumor necrosis factors α
IL6: Interleukin 6
NHANES: National Health and Nutrition Examination Survey
IDPN: Intra-dialytic parenteral nutrition
CT: Computed tomography
MRI: Magnetic resonance imaging
DEXA: Dual energy x-ray absorptiometery
