# Supplementary material for: Supervised oral protein supplementation during dialysis in patients with elevated C-reactive protein levels: a two phase, longitudinal, single center, open labeled study
Source: BMC Nephrol. 2015 Jun 23;16:87. doi: 10.1186/s12882-015-0070-0 (PMC4477490; doi:10.1186/s12882-015-0070-0)

**Appendix Table 1 - Effects of protein supplementation on nutritional parameters in sub-groups defined by baseline PNA**

|  | Δ interventional phase (95% CI), p values | |
| --- | --- | --- |
|  | PNA<1 (g/kg/day)  N=22* | PNA ≥ 1 (g/kg/day)  N=28* |
| MAMC (cm) | 0.45 (-0.65, 1.56), p=0.40 | -0.15 (-1.15, 0.85), p=0.76 |
| Plasma Albumin (g/dL) | -0.02 (-0.13, 0.09), p=0.70 | -0.01 (-0.14, 0.11), p=0.86 |
| BMI (kg/m²) | -0.09 (-0.36, 0.19), p=0.53 | -0.24 (-0.64, 0.16), p=0.23 |
| PCS | -1.28 (-6.34, 3.79), p=0.60 | -2.32 (-6.41, 1.76), p=0.25 |
| MCS | 0.49 (-3.99, 4.97), p=0.82 | -0.92 (-3.70, 1.87), p=0.51 |

*Based on baseline PNA values

**Appendix Table 2 - Effects of protein supplementation on nutritional parameters in sub-groups defined by baseline plasma albumin**

|  | Δ interventional phase (95% CI), p values | |
| --- | --- | --- |
|  | Plasma albumin <4 (g/dL)  N=33* | Plasma albumin ≥ 4 (g/dL)  N=17* |
| PNA (g/kg/day) | 0.18 (0.06, 0.30), p=0.004 | 0.04 (-0.14, 0.23), p=0.61 |
| MAMC (cm) | 0.04 (-0.87, 0.96), p=0.92 | -0.06 (-1.24, 1.13), p=0.92 |
| BMI (kg/m²) | -0.13 (-0.42, 0.17), p=0.38 | -0.48 (-0.96, -0.00), p=0.05 |
| SF12- PCS | -0.65 (-4.53, 3.23), p=0.74 | -4.20 (-8.93, 0.53), p=0.08 |
| SF12- MCS | 0.17 (-3.09, 3.43), p=0.92 | -0.42 (-4.09, 3.24), p=0.81 |

*Based on baseline plasma albumin values

**Appendix Table 3 - Effects of protein supplementation on nutritional parameters in sub-groups defined by baseline plasma hsCRP**

|  | Δ interventional phase (95% CI), p values | |
| --- | --- | --- |
|  | Plasma hsCRP <10 (g/dL)  N=17* | Plasma hsCRP ≥ 10 (g/dL)  N=33* |
| PNA (g/kg/day) | 0.10 (-0.09, 0.29), p=0.27 | 0.16 (0.04, 0.27), p=0.009 |
| MAMC (cm) | -1.08 (-1.80, -0.35), p=0.006 | 0.58 (-0.27, 1.44), p=0.18 |
| Plasma Albumin (g/dL) | -0.01 (-0.15, 0.14), p=0.93 | -0.03 (-0.12, 0.06), p=0.55 |
| BMI (kg/m²) | -0.56 (-1.16, 0.05), p=0.07 | -0.09 (-0.36, 0.18), p=0.52 |
| SF12- PCS | -1.07 (-6.75, 4.61), p=0.69 | -1.65 (-5.41, 2.10), p=0.38 |
| SF12- MCS | 0.66 (-2.82, 4.13), p=0.69 | 0.09 (-3.26, 3.45), p=0.95 |

*Based on baseline hsCRP values

**Appendix Table 4 - Provide Gold® Sugar Free 30oz bottles amino acids components**

| Typical amino acids  (g/100g protein) |  |
| --- | --- |
| Alanine | 11.0 |
| Arginine | 8.7 |
| Aspartic Acid | 6.5 |
| Cysteine | 0.2 |
| Glutamic Acid | 11.3 |
| Glutamine | 0.5 |
| Glycine | 26.4 |
| Histidine* | 1.4 |
| Hydroxylysine | 0.7 |
| Hydroxyproline | 12.9 |
| Isoleucine* | 1.5 |
| Leucine* | 3.4 |
| Methionine* | 4.2 |
| Phenylalanine* | 1.2 |
| Proline | 2.4 |
| Serine | 15.1 |
| Threonine* | 3.6 |
| Tryptophan* | 3.3 |
| Tyrosine | 0.8 |
| Valine* | 0.3 |
| * Essential Amino Acid | 2.7 |

**Appendix Figure 1 –Provide Gold® Sugar Free 30oz bottles nutrition facts**


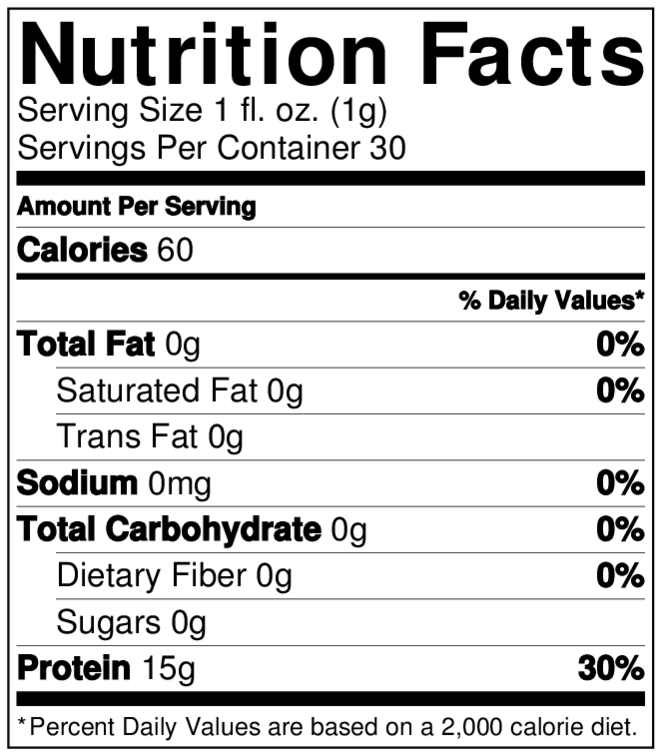

Supplement: Supplementary file 1 — Supplemental materials. Appendix Table 1 - Effects of protein supplementation on nutritional parameters in sub-groups defined by baseline PNA. Appendix Table 2 - Effects of protein supplementation on nutritional parameters in sub-groups defined by baseline plasma albumin. Appendix Table 3 - Effects of protein supplementation on nutritional parameters in sub-groups defined by baseline plasma hsCRP. Appendix Table 4 - Provide Gold® Sugar Free 30oz bottles amino acids components. Appendix Figure 1 –Provide Gold® Sugar Free 30oz bottles nutrition facts. [file 12882_2015_70_MOESM1_ESM.docx]
